# Modeling passenger comfort in turboprop aircraft using objective measures

**DOI:** 10.3233/WOR-230700

**Published:** 2027-08-01

**Authors:** Yu (Wolf) Song, Anna S. Reichherzer, Xinhe Yao, Gerbera Vledder, Britta Herbig, Michael Bellmann, Victor Norrefeldt, Peter Vink, Neil Mansfield

**Affiliations:** 1 Faculty of Industrial Design Engineering, Delft University of Technology, Delft, The Netherlands; 2 Institute and Clinic for Occupational, Social and Environmental Medicine, University Hospital, LMU, Munich, Germany; 3 Fraunhofer Institute for Building Physics IBP, Fraunhoferstrasse 10, 83626 Valley, Germany; 4 Institut für technische und angewandte Physik GmbH, Oldenburg, Germany; 5 Department of Engineering, Nottingham Trent University, Nottingham, UK

**Keywords:** Comfort, discomfort, model, turboprop

## Abstract

**Background:**

A quantitative comfort model will aid in evaluating comfort levels of various target groups before the actual flight of an airplane. However, constructing the model is always a challenge due to the complexity of the phenomenon.

**Objectives:**

In this paper, we present quantitative comfort models to predict the (dis)comfort of passengers flying with turboprops based on objective measures.

**Methods:**

Ninety-seven participants took part in two experiments conducted during real flights, during which forty of them had environmental and personal factors recorded using (self-developed) measurement tools. The collected data were analyzed to model the relations between objective measures and subjective feelings.

**Results:**

Two preliminary models based on gradient boosting regression were developed. The models were able to predict the changes in comfort and discomfort of individual passengers with an accuracy of 0.12±0.01 and 0.21±0.01 regarding normalized comfort and discomfort scores, respectively. Additionally, contributions of different factors were highlighted.

**Conclusion:**

The outcomes of the models show that we took a step forward in modeling the human comfort experience using objective measurements. Anthropometry (including age), seat positions, time duration, and row (noise) emerged as leading factors influencing the feeling of (dis)comfort in turboprop planes.

## 1. Introduction

In 2022, Clean Aviation announced its ambitious target of decreasing aircraft greenhouse gas emissions by no less than 30% by 2030, aiming at climate-neutral aviation by 2050 [1]. While fuel, propulsion systems, lightweight materials, and structures have attracted a lot of attention, the comfort of passengers is another important factor for an environmentally friendly and enjoyable journey [2].

The subjective (dis)comfort feelings of passengers involve complex constructs [3]. Researchers have begun to interpret this phenomenon using a series of qualitative models [3–8], and it has been proposed that the factors influencing comfort can be categorized as users’ backgrounds, the physical properties of their bodies, their expectations, the (social) environment(s), the product(s) they are using, the interactions between the users and the product/environment, and the duration of the use [7].

Turboprop airplanes play a significant role in promoting more sustainable aviation, as they consume 10–60% less fuel compared to regional jet flights [9]. However, turboprop passengers may experience different levels of comfort compared to those in jet aircraft. For instance, according to Bouwens et al. [10], the comfort feelings of passengers in jet engine airplanes depend on various factors such as seating, noise, lighting, temperature, vibrations, and odor, ranked from high to low importance. On the other hand, Vink et al. discovered that noise is the primary contributor to discomfort in turboprop aircraft [11]. This is reflected in noise measurements showing that the average cabin noise level in an Airbus A350 is approximately 74.9 dB(A) [12], while in an ATR 72, it can reach over 80 dB(A) [13]. Future generations of turboprop aircraft need to provide a better comfort experience to be widely accepted by passengers and operated by airlines.

In interior design for the new generation of turboprops, a quantitative model for passenger comfort and discomfort is essential. This includes optimizing space utilization and crafting ergonomic seat designs. However, although the factors influencing comfort are relatively clear, constructing a model for individuals in the cabin and highlighting the effects of different parameters remains challenging due to the complexity of the environment and the differences among individuals. Among different modeling methods, (linear) regression models were often used to describe collective passenger behavior [14]. Similarly, structural equation models were used as well for incorporating more factors [15]. For a better prediction of individual (dis)comfort, data-driven methods, e.g., machine learning, have been highlighted for their ability to address various factors of complex phenomena. For instance, Zhao et al. used data-driven methods in modeling thermal comfort of users [16], and an Improved Particle Swarm Algorithm – Supported Vector Machine (IPSO-SVR) method was used to predict comfort of pilot seats based on pressure data [17]. However, when employing a data-driven approach, the availability of a valid (large) dataset specific to the target group is crucial.

In the European project COMFDEMO, we modeled the (dis)comfort experience of passengers seated in the turboprop aircraft cabin. This paper outlines the experiment conducted for modeling, the data collection tools, and the modeling tool, and presents the initial comfort models for passengers. Cross-validation results suggest the potential, along with a notable degree of uncertainty, in using the model to predict comfort levels of individuals based on objective measures of users, users’ background, the environment, the products, as well as the duration of use.

## 2. Materials & methods

**Figure 1.**
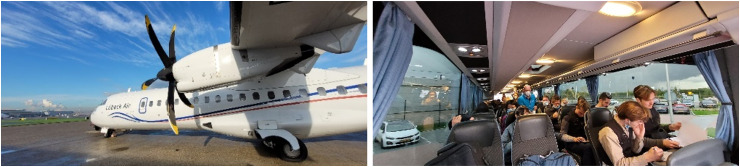
Left: The ATR72-500 plane from Lubeck Air, Right: Participants on the way from the airport with Jacket.

An experiment was carried out with two flights at Rotterdam Airport, one in the morning and another in the afternoon, each lasting about 70 minutes. The ground temperature of the day in the airport was 12°C and the relative humidity was approximately 78% on the ground. The flights were conducted using an ATR72-500 turboprop (Fig. 1), with a (cruising) flight altitude at 17,000 feet, and the cabin pressure was around 900 hPa during the cruising stage [13].

### 2.1. Measurement tools

**Figure 2.**
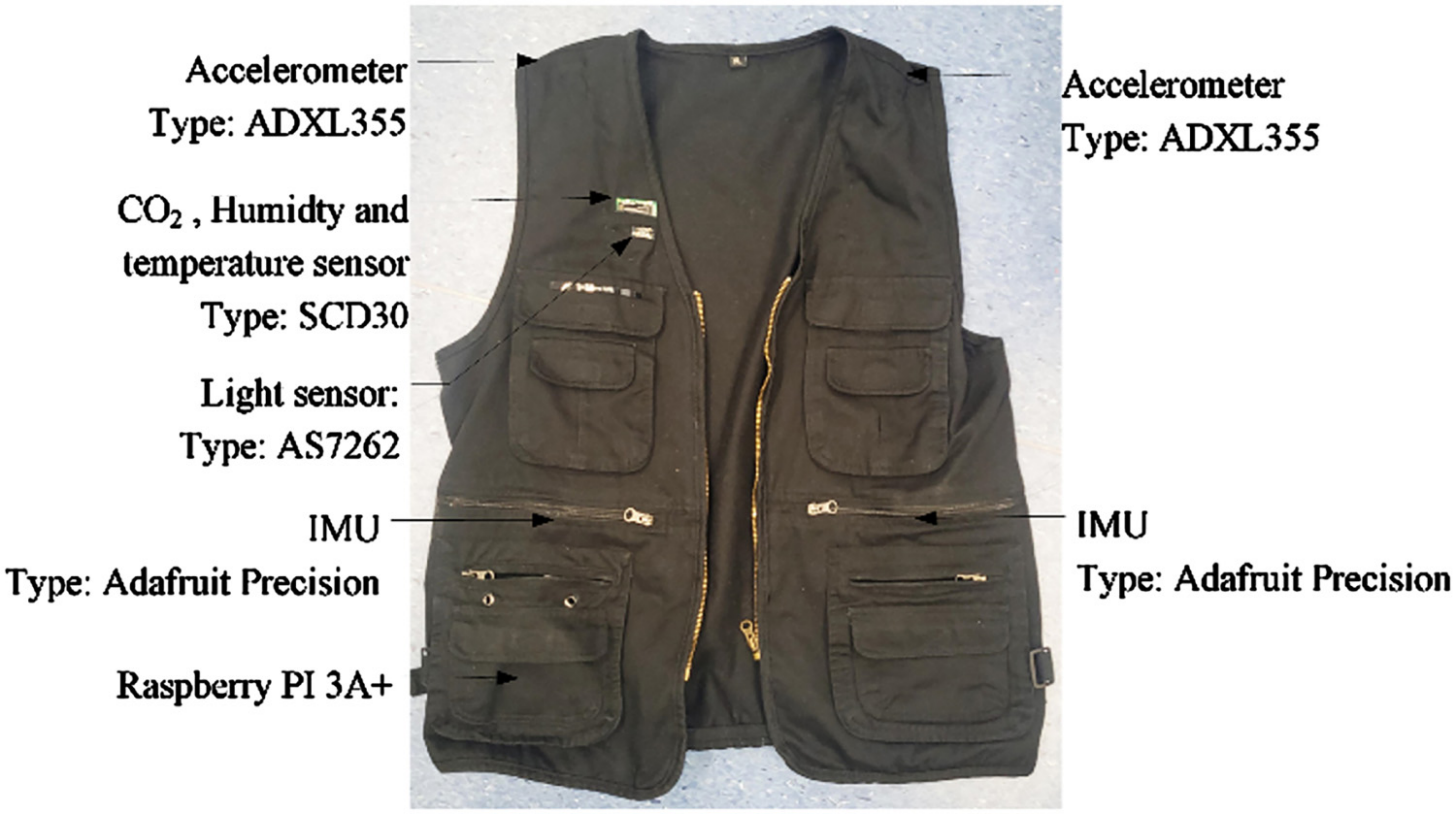
An example of the 20 measurement Jackets.

A series of tools were used to log environmental and personal variables during the flight. For instance, noise levels in different rows were documented using a Bruel & Kjaer 2270 sound level meter positioned in the middle of each row [13]. A wearable measurement tool, called the *Jacket*, was developed to gather data on passengers’ physical movements and local environmental parameters [18]. Specifically, on each side (left/right) of the trunk, the (contra)lateral, superior/inferior, and anterior/posterior movements of the shoulder and waist were measured by an ADXL355 accelerometer and an Adafruit Precision IMU, respectively. CO_2_ levels, temperature, and humidity were logged by an SCD30 sensor, and the light spectrum was recorded by an AS7262 sensor at the right chest position. Twenty jackets in four different sizes were manufactured, and Fig. 2 shows one of them.

### 2.2. Participants

**Figure 3.**
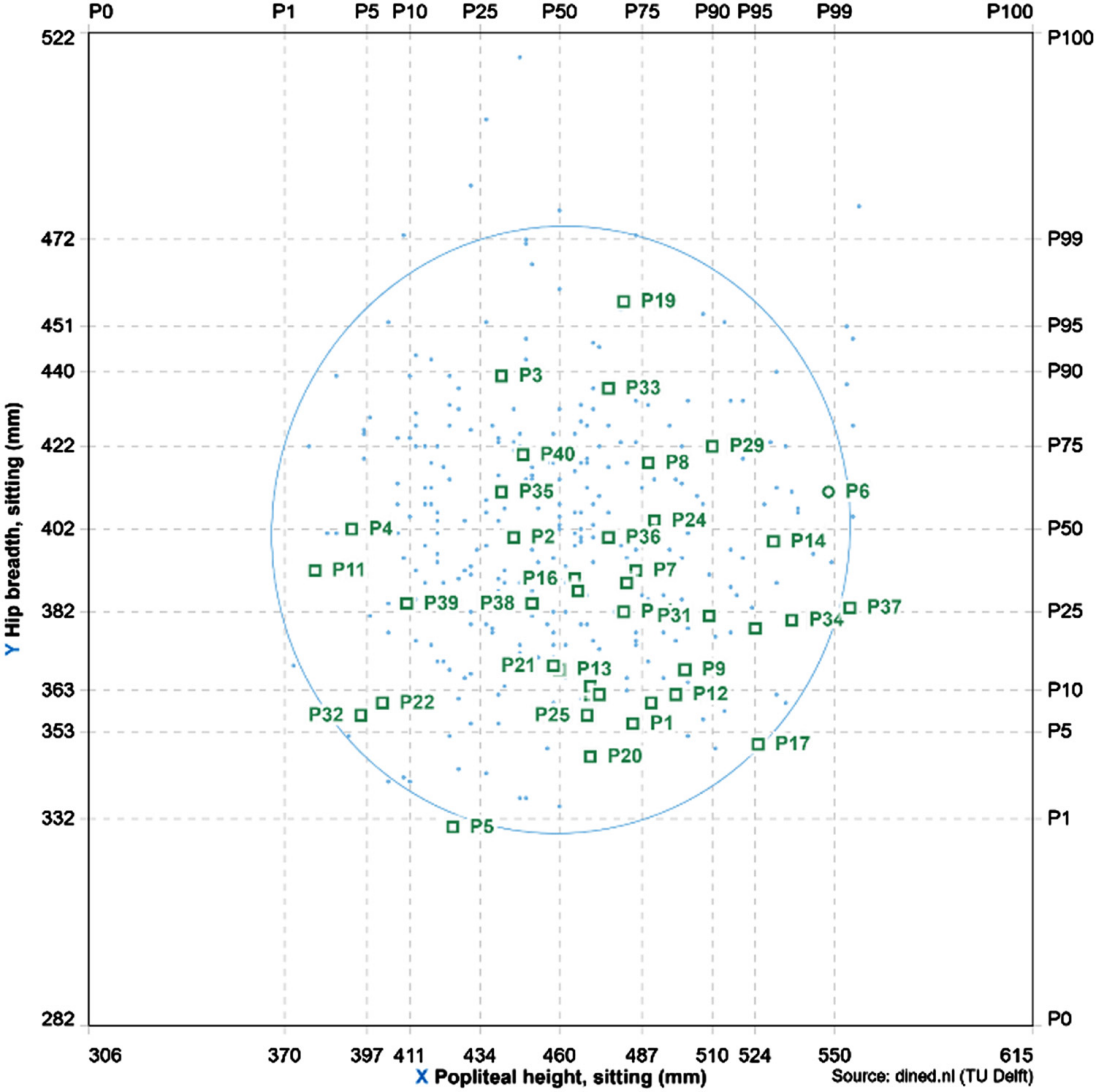
The distribution of anthropometric measurements of the 40 participants.

Among all participants on each of the two flights, 20 of them were chosen to wear the measurement *Jacket*, resulting in a total of 40 datasets. The mean age of the 40 participants is 35.15±15.08 years old, with a mean stature of 174.2±8.6 cm. The mean body weight is 74.0±13.9 kg. In terms of Sex distribution, there are 26 males and 14 females. During recruitment, we utilized self-reporting [19] as well as on-site measurement methods to minimize the specificity of the population in relation to key anthropometric measurements associated with (dis)comfort. Figure 3 shows the distribution of hip-breadth(width) regarding popliteal height of the forty participants who wore the *Jackets*.

**Figure 4.**
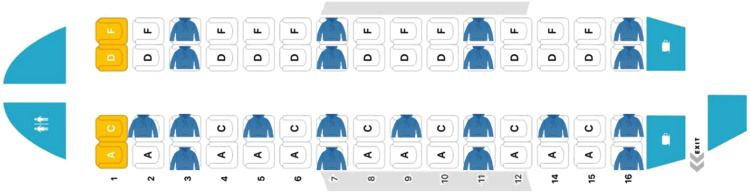
The location of Jackets (worn by participants) in the plane for both morning and afternoon flights. The number “13” was not used in row numbers for this plane.

In the seating arrangement of these 40 participants, the consortium shortlisted several options, including random distributions. Based on the available number of *Jackets* and the cabin layouts of the specific ATR72-500 turboprop, it was decided that a relatively uniform distribution of *Jackets* across the left-right and fore-aft directions in the cabin would be most helpful in understanding the influence of environmental parameters on the passengers. In the proposed layouts, participants wore *Jackets* in Rows 3, 7, 11, and 16 (Row 13 was unavailable on the plane). Furthermore, participants occupying Seats 2 C, 5 C, 9 C, and 14 C were also wearing *Jackets*, as illustrated in Fig. 4. Among these 20 designated seats, participants had the freedom to select their seat positions according to their preferences.

### 2.3. Protocols

Upon signing the informed consent, participants received a briefing about the procedure and selected a *Jacket* that corresponded to their body size. Once onboard the aircraft, they completed questionnaires on various (dis)comfort aspects at different flying stages [20], including taxiing, takeoff/climbing, cruising, descending, and taxiing after landing [21].

### 2.4. Data analysis methods

Objective measurement data gathered from various measurement tools underwent pre-processing. Table 1 lists the category, specific measured parameters, measurement locations, and the correction methods applied to the collected raw data.

**Table 1.** Data types and collection methods.

Category	Factors be measured	Measurement location	Correction methods
Time	Time	Each Jacket	Synchronized by peak CO_2_ levels
Temperature & Air quality	Temperature	Each Jacket	No
	CO_2_ levels	Each Jacket	Corrected by equations
	Humidity	Each Jacket	No
Vibroacoustic	Sound pressure levels (SPLs)	Aisle of each row	No
Layout	Row	By seat	14 15 and 16 changed to 13, 14 and 15
	Seat (A, C, D or F)	By seat	An extra virtual seat was inserted between C and D to simulate the aisle
Flights	Morning/ Afternoon	By flight	Changed to 0 or 1
Light intensity	Red	Each Jacket	No
	Orange	Each Jacket	No
	Yellow	Each Jacket	No
	Green	Each Jacket	No
	Blue	Each Jacket	No
	Violet	Each Jacket	No
Ergonomics	Sex	Measured before flight	Changed to 0 or 1
	Age	Measured before flight	No
	Stature	Measured before flight	No
	Body mass	Measured before flight	No
	Popliteal height	Measured before flight	No
	Buttock popliteal depth	Measured before flight	No
	Hip width	Measured before flight	No
Physical Posture changes/motion	Left shoulder	Each Jacket	Change to human physical activities using the sensor motion package [22]
	Right shoulder	Each Jacket	
	Left waist	Each Jacket	
	Right waist	Each Jacket	

Among all the data, data from Jacket No. 5 (Seat 2C), Jacket No. 10 (Seat 3D) in the morning, and Jacket No. 9 (Seat 11C) and No. 18 (Seat 5C) in the afternoon were missing, most likely due to power management issues of the embedded system. The slight variations in the starting times of the jackets (1–2 minutes) were minimized by synchronizing the CO_2_ concentration level peaks just before engine start. Physical activities of the left/right shoulders and left/right waists were extracted from the four accelerometers and then pre-processed using the sensor motion package [22] for the three axes, respectively. CO_2_ concentration levels were corrected by the pressure measured during our flights as reported in Müller et al. [13] with the following Equations where *t* is the timestamp of the records and ${CO}_{2}^{reading}$ is the original readings of the sensor.

$$\begin{array}{cccc} & CO_{2}^{reading} & t=0\;∼480 & \mathrm{Taxiing}\\ & CO_{2}^{reading}*\left(1+\frac{t-480}{720}*\left(1/0.9-1\right)\right) & t=480\;∼1200s & \mathrm{Takeoff}/\mathrm{climbing}\\ CO_{2}^{correct}\;= & CO_{2}^{reading}*\frac{1}{0.9} & t=1200\;∼2580\;s & \mathrm{Cruising}\\ & CO_{2}^{reading}*\left(1/0.9-\frac{t-2580}{600}*\left(1/0.9-1\right)\right) & t=2580\;∼3180\;s & \mathrm{Descending}\\ & CO_{2}^{reading} & t>3180\;s & \mathrm{Taxiing} \end{array}$$

All measurement data were scaled to the range of {0, 1} using the min–max scaler [23]. Concurrently, the questionnaire data on comfort and discomfort were normalized using the min–max scaler as well. It is worth noting that through this process, the scores on comfort and discomfort were changed to reflect changes in comfort and discomfort. Linear interpolation methods were employed to sample all parameters and comfort scores at 60-second intervals. Correlations between each parameter and the (dis)comfort scores were computed first to highlight important parameters. Parameters with correlations to (dis)comfort (*p* < 0.1) were selected as inputs for training two models, establishing relations with comfort and discomfort scores. The most significant contributors to comfort and discomfort were identified by assessing their contributions using the permutation importance method [24].

## 3. Results

**Figure 5.**
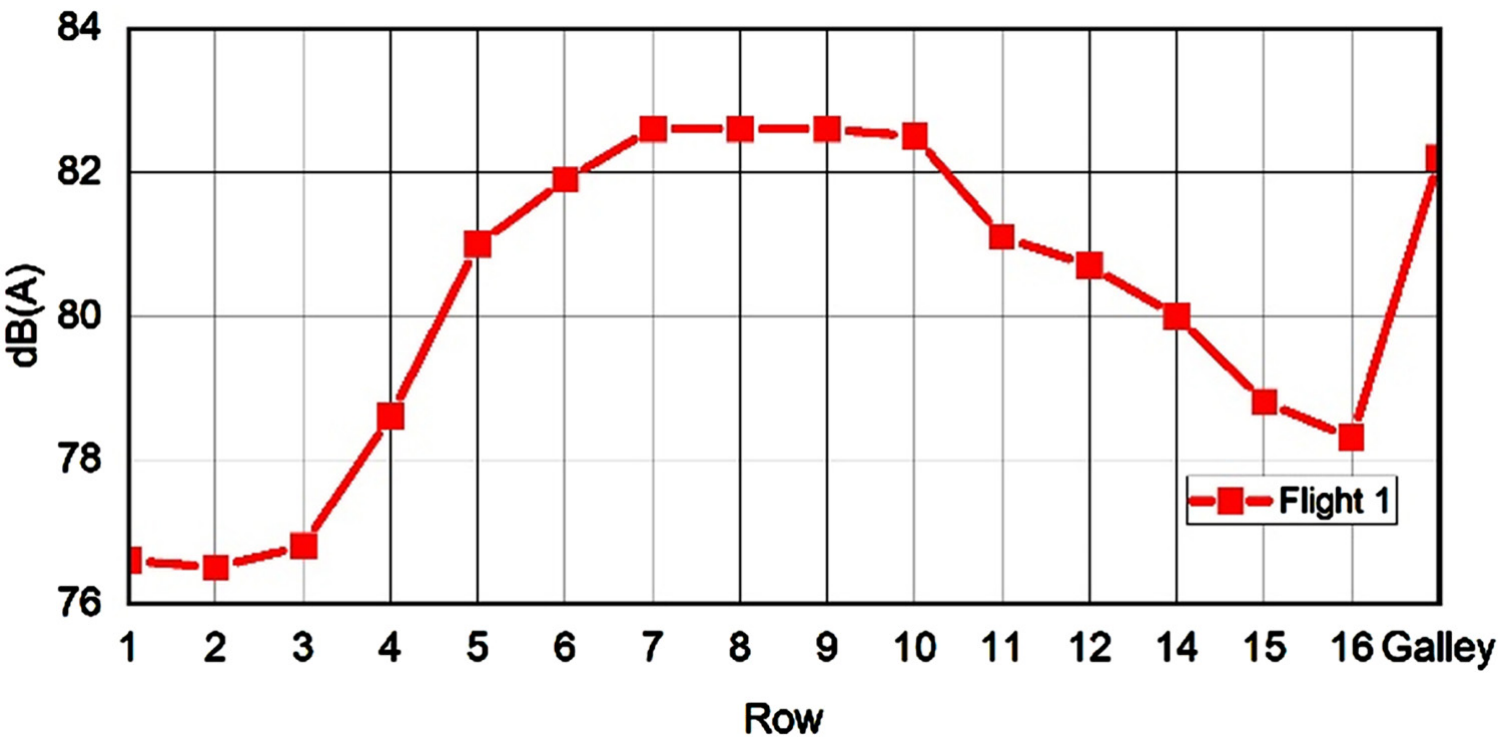
Cabin noise levels, measured in the aisle, courtesy of [13].

Subjective and objective data collected from questionnaires and different measurement tools were extracted and stored for later analysis. Figure 5 presents the measured noise levels across the turboprop plane.

Table 2 displays all parameters along with p-values of their correlations with (dis)comfort scores over time. In total, 18 comfort and 16 discomfort parameters exhibit significant correlations (*p* < 0.01). To ensure that all (dis)comfort-related factors were included, a threshold of *p* = 0.1 was utilized to select parameters for modeling the comfort experience. This increases the number of parameters to 26 for comfort and 19 for discomfort. We did not set any thresholds for correlation values, as we anticipate nonlinear relationships between objective measurements and subjective feelings. Instead, we will identify the influence of factors using the models and the permutation importance method.

**Table 2.** Parameters and their correlations with (dis)comfort.

Parameters	Parameter Index	Correlations with comfort	*P* value of correlations with comfort	Correlations with discomfort	*P* value of correlations with discomfort
Time	P1^#^^Δ^	–0.18	***p* < 0.01**	0.07	***p* < 0.01**
Row	P2^#^^Δ^	–0.14	***p* < 0.01**	0.17	***p* < 0.01**
Seat (A, C, D or F)	P3^#^^Δ^	0.06	***p* < 0.01**	0.3	***p* < 0.01**
Morning or Afternoon	P4^#^	0.05	***p* < 0.01**	0.03	0.2
Gender	P5^#^^Δ^	–0.04	**0.04**	0.12	***p* < 0.01**
Age	P6^#^^Δ^	0.03	**0.09**	–0.16	***p* < 0.01**
Stature	P7^#^^Δ^	0.08	***p* < 0.01**	0.05	***p* < 0.01**
Body Mass	P8^#^^Δ^	0.22	***p* < 0.01**	0.16	***p* < 0.01**
Popliteal height	P9^#^	0.04	**0.03**	0.03	0.21
Buttock popliteal depth	P10^#^^Δ^	0.13	***p* < 0.01**	0.13	***p* < 0.01**
Hip width	P11^#^^Δ^	0.26	***p* < 0.01**	0.13	***p* < 0.01**
Noise	P12^Δ^	0.01	0.73	0.14	***p* < 0.01**
Right shoulder X-(contra) Lateral	P13^#^	–0.15	***p* < 0.01**	0.02	0.23
Right shoulder Y-Anterior/Posterior	P14^Δ^	0.01	0.76	–0.16	***p* < 0.01**
Right shoulder Z-Superior/Inferior	P15^#^^Δ^	0.07	***p* < 0.01**	–0.04	**0.03**
Left shoulder X-(contra)Lateral	P16^#^	–0.16	***p* < 0.01**	–0.03	0.11
Left shoulder Y-Anterior/Posterior	P17^#^^Δ^	–0.17	***p* < 0.01**	–0.07	***p* < 0.01**
Left shoulder Z- Superior /Inferior	P18^#^	0.19	***p* < 0.01**	–0.01	0.58
Right Waist X-(contra)Lateral	P19^#^	–0.04	**0.04**	0.03	0.19
Right Waist Y- Superior /Inferior	P20	0.02	0.25	–0	0.82
Right Waist Z-Anterior/Posterior	P21^#^	–0.08	***p* < 0.01**	–0.02	0.44
Left Waist X-(contra)Lateral	P22^#^	0.07	***p* < 0.01**	–0.01	0.77
Left Waist Y-Superior/Inferior	P23^#^	0.05	**0.02**	–0.03	0.16
Left Waist Z-Anterior/Posterior	P24^Δ^	0.02	0.26	–0.04	**0.03**
CO_2_ level	P25^#^	0.07	***p* < 0.01**	–0.03	0.11
Temperature	P26^#^	–0.05	**0.02**	–0.01	0.53
Humidity	P27^#^^Δ^	0.22	***p* < 0.01**	–0.12	***p* < 0.01**
Red light intensity	P28^#^^Δ^	–0.05	**0.01**	0.03	**0.09**
Orange light intensity	P29^#^	–0.05	**0.02**	–0.03	0.18
Yellow light intensity	P30^Δ^	–0.01	0.62	–0.09	***p* < 0.01**
Green light intensity	P31^Δ^	–0.02	0.25	–0.08	***p* < 0.01**
Blue light intensity	P32^Δ^	–0.02	0.27	–0.07	***p* < 0.01**
Violet intensity	P33^#^	–0.09	***p* < 0.01**	0.02	0.26

*p-values in bold indicate that the parameter is selected. ^#^Parameter is selected as a predictor of comfort. ^Δ^Parameter is selected as a predictor of discomfort.

The identified parameters were used as inputs for two Gradient Boosting Regression models *G*_*c*_ and *G*_*d*_ [25] where the comfort and discomfort scores were used as the outputs as:

*Comfort* = *G*_*c*_ (*P*_1_ ⋯ *P*_11_, *P*_13,_*P*_15_ ⋯ *P*_19_, *P*_21_ ⋯ *P*_23_, *P*_25_ ⋯ *P*_29_, *P*_33)_

and

*Discomfort* = *G*_*d*_ (*P*_1_ ⋯ *P*_3_, *P*_5_ ⋯ *P*_8_, *P*_10_ ⋯ *P*_12_, *P*_14,_*P*_15_, *P*_17_, *P*_24_, *P*_27_, *P*_28_, *P*_30_ ⋯ *P*_32_)

Here model, *G*_*c*_ is used to predict changes in passengers’ comfort levels, and *G*_*d*_ for predicting changes of discomfort levels. Both models were trained using the collected 36 datasets and a self-developed Python program. The 5-fold cross-validation method was utilized to validate the accuracy of both models [26]. The results of cross validation indicated that the root mean square errors (RMSEs) of the model *G*_*c*_ for predicting changes of comfort and *G*_*d*_ for changes of discomfort were 0.12±0.01 and 0.21±0.01, respectively. This suggests that the RMSEs represent a variation of 12% in comfort changes and 21% in discomfort changes, considering that the (dis)comfort scores were normalized using the min–max scaler within the domain of {0, 1}.

**Figure 6.**
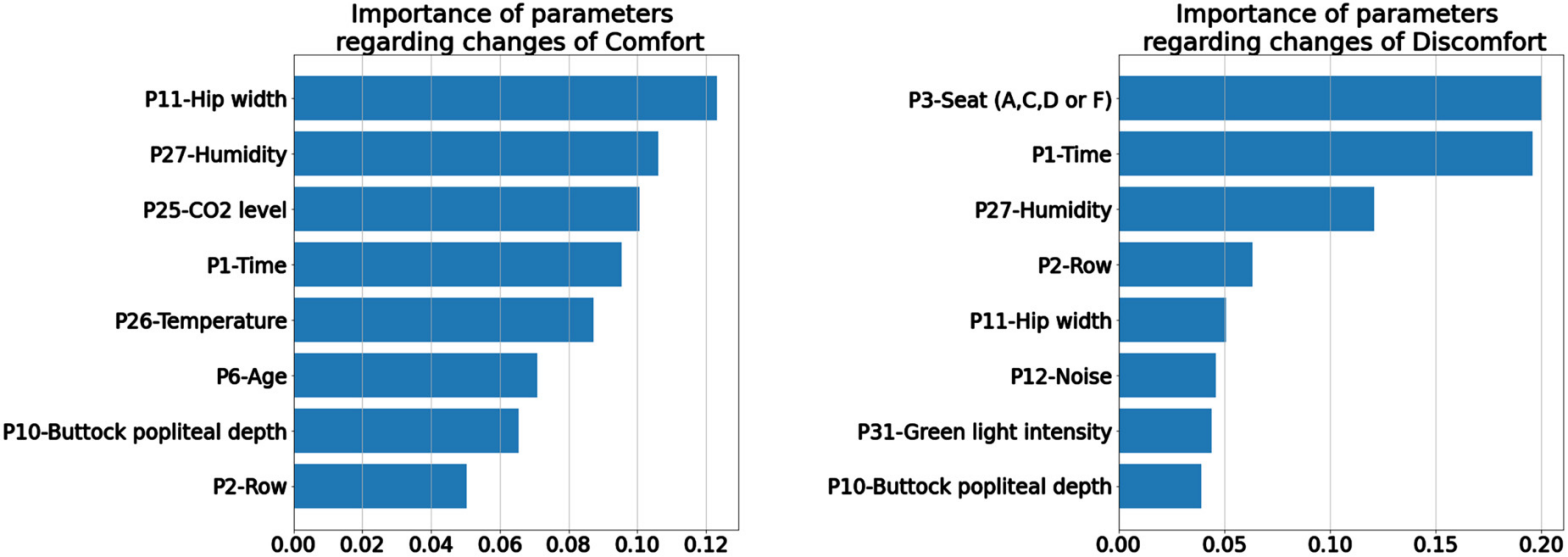
The importance of factors regarding (dis)comfort (left comfort, right discomfort, horizontal axes represent the amplitude of the contribution).

Using both models and the permutation importance method, we ranked the contributions of different parameters concerning the models’ outputs. It was found that for comfort, hip width was the most important factor, followed by humidity, CO_2_ level, time, temperature, age, buttock popliteal depth, and row number, which was closely associated with noise levels. Conversely, for discomfort, the prominent factors were seat location (windows/aisle), time, humidity, row, hip width, noise levels, green light intensity, and buttock popliteal depth. The amplitudes of these contributions are presented in Fig. 6.

## 4. Discussion

### 4.1. The quantitative model and accuracy

In this paper, we collected environmental and passengers’ data from actual turboprop flights and developed two quantitative models for predicting comfort and discomfort, respectively. To minimize reliance on sensor accuracy and subjective perceptions of comfort and discomfort, we employed the min–max scaler to transform parameters and predictions into relative values, such as predicting changes in (dis)comfort levels. Our model incorporates 26 parameters for predicting changes in comfort levels and 19 parameters for changes in discomfort levels. Using collected 36 data sets, our models are able to predict the changes of comfort and discomfort with an RMSE of 12% and 21% , respectively.

In a laboratory setup, Aggarwal et al. collected data on noise and vibration and developed a linear model to predict the comfort level of passengers with an RMSE of 8.5% [14]. Zhang et al. used 162 sets of pressure data to predict the comfort of subjects in a pilot seat, and the prediction accuracy of their IPSO-SVR model was 94% in an 80% training and 20% testing setup [17]. Zhao et al. reviewed about 40 articles on thermal comfort models, and they found the prediction accuracy of the algorithm using decision tree can be more than 90% [16]. Compared to these results, the accuracies of the proposed models are not high. Several potential reasons contribute to this outcome: 1) Data used for the proposed models were collected on real flights instead of a controlled environment, incorporating more noise in the data. 2) The dataset comprised only 36 sets (with 4 missing). Acquiring more data could potentially enhance the model’s accuracy. 3) We utilized the min–max scaler for data normalization, and instead of predicting absolute values, the proposed models predict the changes of (dis)comfort. 4) Constant factors during the flight, such as seat width, were not included in the model. Further investigation into both data pre-processing techniques, e.g., using the z-score method [27], and modeling methods, e.g., DNN and network pruning [28], might yield improved results.

### 4.2. Comfort vs discomfort factors

Further analysis of the effect of different parameters reveals that the sensation of comfort results from the interplay of psychological, social and physical aspects in humans. Long-term sitting leads to rising levels of discomfort, highlighting the importance of seating time on both the feelings of comfort and discomfort [29]. Despite over 99% of the population being fitted by modern airplane seats, individuals still desire greater space for movement over time [30]. While the dimensions of all seats were the same in our experiments, this desire was reflected in the significance of anthropometric measures such as hip-width and buttock popliteal depth, both of which restricted the freedom of movements of passengers in the seat. Additionally, older individuals might prioritize different aspects of comfort compared to younger individuals [31], and age emerged as an important determinant of comfort feeling, despite it has lower impact on discomfort.

Environmental factors influence the feeling of (dis)comfort. Passengers in the aisle and the window seats experienced different levels of discomfort. Furthermore, we observed that exposure to green light may also influence feelings of discomfort. The row number exhibited a strong correlation with noise in the ATR 72-500 (Fig. 5), underscoring noise’s impact on passenger comfort in turboprop airplanes. Our findings also suggested that temperature and humidity were important factors for comfort, while humidity was also crucial for discomfort. We also noticed that CO_2_ levels affect comfort, however Herbig et al. suggested that CO_2_ levels were not correlated with comfort/discomfort in their randomized clinical trial [32]. In addition, CO_2_ concentrations and humidity were very homogeneous in the cabin [33]. In our experiment, the recorded CO_2_ and humidity levels in the local environment might be correlated with the amount of Volatile Organic Compounds (VOCs) emitted by humans [34]. It was conceivable that humidity and CO_2_ merely served as an indicator of VOC presence, which, in turn affects the participants’ perception of comfort.

Though most factors that influence the levels of comfort and discomfort are similar, there are certain differences: 1) the contribution of factors to the comfort tends to be smaller than the contribution to discomfort, which can be reflected in the smaller amplitude on the horizontal axis of Fig. 6; 2) age plays a vital role in comfort perception. Both of these observations indicate a more complicated construct of the feeling of comfort, aligning with literature suggesting that comfort encompasses more psychological constructs [7]. In contrast, discomfort predominantly arises from physical interactions between users and their environment or products. Factors such as seat positions, time, and anthropometry emerge as dominant discomfort factors, consistent with existing literature [3, 29].

### 4.3. Limitations

Ethical considerations prevented the measurement of noise in the user’s micro-environment. Technical challenges also hindered the measurement of micro-environmental vibration for each subject. As a result, the model does not incorporate these factors, despite their significance according to the literature [14]. Moreover, the specific ATR72-500 has a relatively large seat pitch of 34 inches, potentially influencing the importance of other anthropometric measures like stature and popliteal height.

## 5. Conclusion

This study introduces two models aimed at predicting passenger (dis)comfort dynamics within the context of turboprop travel. Our findings represent advancements in quantifying the human comfort experience through the utilization of objective measurements collected during real flights. Despite limitations posed by a constrained dataset, the proposed models demonstrated reasonable predictive accuracy, achieving RMSEs of 0.12±0.01 and 0.21±0.01 for predicting changes in normalized comfort and discomfort, respectively.

Using the permutation importance method, we identified critical parameters influencing the predictive outcomes. Anthropometric factors, including age, hip-width, and buttock popliteal depth, emerged as pivotal determinants of (dis)comfort. Besides, environmental variables such as humidity, CO_2_ levels (linked to VOC concentrations in our study), temperature, seat positioning, row allocation, noise levels, and green light intensity were identified as primary contributors to passenger discomfort. In addition to anthropometry and environmental factors, our analysis underscores the critical role of time in shaping the (dis)comfort experience. This insight lays the groundwork for enabling explainable-AI-based minimum viable sensing methods for real-time prediction of (dis)comfort of passengers [35]. Furthermore, this knowledge can contribute to the development of personalized interventions [36] aimed at optimizing aircraft design for improved passenger well-being.
